# Thermometry of Red Blood Cell Concentrate: Magnetic Resonance Decoding Warm Up Process

**DOI:** 10.1371/journal.pone.0057931

**Published:** 2013-02-28

**Authors:** Gert Reiter, Ursula Reiter, Thomas Wagner, Noemi Kozma, Jörg Roland, Helmut Schöllnast, Franz Ebner, Gerhard Lanzer

**Affiliations:** 1 Healthcare Sector, Siemens AG, Graz, Austria; 2 Department of Radiology, Medical University of Graz, Graz, Austria; 3 Department of Blood Group Serology and Transfusion Medicine, Medical University of Graz, Graz, Austria; 4 Healthcare Sector, Siemens AG, Erlangen, Germany; University of Maryland, United States of America

## Abstract

**Purpose:**

Temperature is a key measure in human red blood cell concentrate (RBC) quality control. A precise description of transient temperature distributions in RBC units removed from steady storage exposed to ambient temperature is at present unknown. Magnetic resonance thermometry was employed to visualize and analyse RBC warm up processes, to describe time courses of RBC mean, surface and core temperatures by an analytical model, and to determine and investigate corresponding model parameters.

**Methods:**

Warm-up processes of 47 RBC units stored at 1–6°C and exposed to 21.25°C ambient temperature were investigated by proton resonance frequency thermometry. Temperature distributions were visualized and analysed with dedicated software allowing derivation of RBC mean, surface and core temperature-time courses during warm up. Time-dependence of mean temperature was assumed to fulfil a lumped capacitive model of heat transfer. Time courses of relative surface and core temperature changes to ambient temperature were similarly assumed to follow shifted exponential decays characterized by a time constant and a relative time shift, respectively.

**Results:**

The lumped capacitive model of heat transfer and shifted exponential decays described time-dependence of mean, surface and core temperatures close to perfect (mean *R^2^* were 0.999±0.001, 0.996±0.004 and 0.998±0.002, respectively). Mean time constants were *τ*_mean_ = 55.3±3.7 min, *τ*_surface_ = 41.4±2.9 min and *τ*_core_ = 76.8±7.1 min, mean relative time shifts were Δ_surface_ = 0.07±0.02 and Δ_core_ = 0.04±0.01. None of the constants correlated significantly with temperature differences between ambient and storage temperature.

**Conclusion:**

Lumped capacitive model of heat transfer and shifted exponential decays represent simple analytical formulas to describe transient mean, surface and core temperatures of RBC during warm up, which might be a helpful tool in RBC temperature monitoring and quality control. Independence of constants on differences between ambient and storage temperature suggests validity of models for arbitrary storage and ambient temperatures.

## Introduction

Storage and transport of human red blood cell concentrate (RBC) is subject of a variety of laws and regulations whereas temperature is a key measure in RBC quality control. According to international guidelines, RBC should be stored at 1 to 6°C to mitigate bacterial contamination, slow metabolic processes but maintain RBC viability [1], [2], [3]. During transport RBC core temperature must not exceed the upper temperature limit of 10°C, else units must be used or discharged [4], [5], [6]. To comply with this standard, RBC transport temperature is commonly estimated either by the time a sample is outside of controlled refrigerated storage or via temperature sensitive labels attached to the surface of RBC pouch. Both methods however give only rather crude estimates of RBC temperature: According to the well-established “30 minutes rule in transfusion medicine” RBCs may be returned to 1 to 6°C storage within 30 min as general upper time limit, whereas neither RBC storage and ambient temperature nor sample volume is taken into account [7], [8], [9]. Controlling temperature by temperature indicators or time–temperature integrator units attached to the surface of the RBC pouch implies that surface temperature well represents sample’s core temperature, which can but doesn’t have to be true [10], [11], [12], [13], [14], [15]. Facing worldwide decreased availability of lifesaving RBC challenges a more precise evaluation of correlations between maximum (surface), mean and minimum (core) temperature in RBC units removed from stock to prevent unnecessary wastage of blood components and, if transfused, ensure minimal risk to patients [1], [2], [3], [16], [17].

Magnetic resonance (MR) thermometry represents a unique tool for non-invasive, contactless, three-dimensional assessment of thermal processes. Although temperature monitoring is feasible with various temperature sensitive MR parameters, proton resonance frequency (PRF) temperature mapping is the method of choice for fast, stable and accurate temperature measurements [18], [19], [20]. After calibration, discrete time-series of mean, surface and core temperatures of RBC units during warm up can be derived from PRF images, which allow analytical modelling of warm up processes. As RBC pouch’s surface resistance to heat transfer can be assumed to be larger than the thermal resistance within RBC, time-dependence of RBC units’ mean temperature during warm up should be well described by a lumped capacitance model of heat transfer, with relative mean to ambient temperature differences following an exponential decay [21], [22]. Additionally, similar time-shifted exponential decays might provide adequate descriptions of surface and core temperature time courses during warm up.

The purpose of the present study was 1) to visualize and analyse warm up processes of RBC withdrawn from steady storage conditions by PRF thermometry, 2) to investigate appropriateness of lumped capacitance model-like behaviour of mean, surface and core temperatures during warm up and 3) to determine and analyse corresponding RBC specific parameters with respect to temperature and geometry dependence.

## Materials and Methods

### RBC Collection

59 RBC units (12 units for calibration and 47 RBC units for warm up experiments), discharged from routine stock of the Department of Blood Group Serology and Transfusion Medicine of the Medical University of Graz due to leucocyte or neopterin elevation, were used within the study. Whole blood donations were obtained according to the Austrian regulations for blood donation [23] from healthy volunteer blood donors after informed written consent and collected into triple bags (LCR5 filtration set) containing 63 ml citrate phosphate dextrose (MacoPharma LAB. Pharmaceutiques, Tourcoing, France). After centrifugation at 4000·*g* for 10 min at 20°C, RBC and plasma were separated from buffy coat fraction and transferred into satellite containers using an automated separator (Compomat G4, NPBI, Amsterdam, Netherlands). Within 30 min after separation RBC was leukoreduced using the LCR5 leukoreduction filter (MacoPharma LAB. Pharmaceutiques, Tourcoing, France) and re-suspended in 100 ml of saline adenine glucose mannitol (SAGM containing 900 mg of glucose monohydrate, 877 mg of sodium chloride, 525 mg of mannitol and 16.9 mg of adenine).

### Sample Storage and Temperature Monitoring

RBC units were stored at temperatures between 1 to 6°C (MobiCool W35 12/230V, Zhuhai, China). To exclude short term effects of the cooling system RBC unit’s storage temperature *T*_storage_ was derived from a RFID temperature data logger (SensoTag Siemens HFST-T109, Vienna, Austria, overall accuracy of ±0.5°C) mounted in a reference RBC unit as internal probe [24].

Throughout experiments ambient temperature in the MR investigation room *T*_ambient_ was stabilized by air conditioning to 21.25°C and controlled by RFID temperature data loggers in 5 min time intervals.

### Determination of PRF Thermal Coefficient of RBC

**Figure 1:**
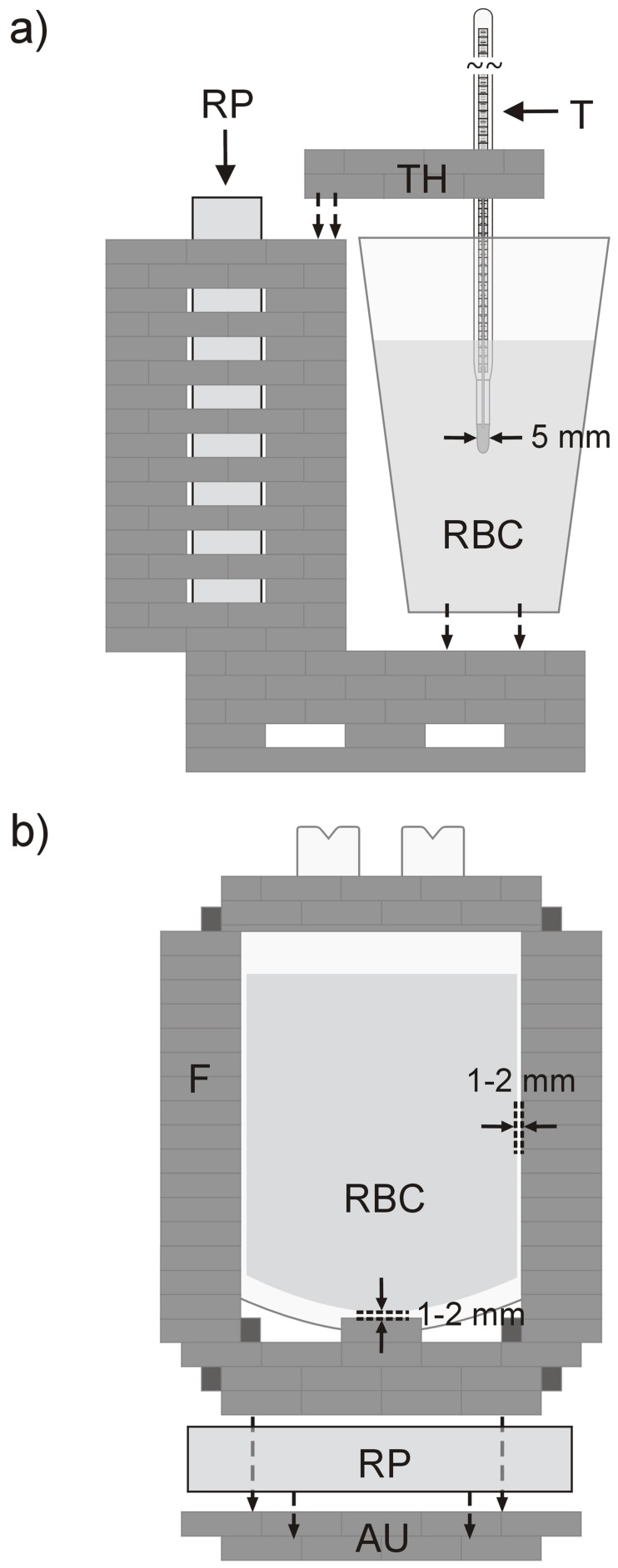
Experimental setup. (a) PRF calibration measurements were performed with RBC stored in a plastic cup. RBC temperature was derived from the calibration thermometer T (tip diameter = 5 mm) positioned at the center of RBC using a thermometer holder TH. A reference phantom RP was positioned in a plastic grid. To avoid thermal cooling of RP by RBC, the wall of the grid next to RBC was isolated by air cushion plastic. (b) RBC units were mounted in upright position in a two-layer plastic frame F (Lego®) with minimum distance of 1–2 mm. For investigation, RBC units were plugged to an adjustment unit AU (Lego®) fixed in a 12 channel head coil adapted with 2 reference phantoms RP kept at MR investigation room temperature *T*_ambient_ throughout experiments.

To derive PRF thermal coefficient of RBC, a series of 12 calibration measurements was performed [25], [26]. A calibration unit (Fig. 1a) build-up of refrigerated RBC, a reference bulb thermometer (Ludwig Schneider Messtechnik, Wertheim, Germany, overall accuracy ±0.2°C) and a reference phantom (5 g agar solved in 500 ml water) kept at ambient temperature to measure non-temperature related phase drifts was positioned in a 1.5 T MR system (Siemens Magnetom Espree, Erlangen, Germany), centered in a 12 channel head coil. After acquisition of reference images, difference phase images covering the thermometer tip with 7 gapless transversal slices and thermometer reference temperatures were measured with 3 min pause time for RBC temperatures between 1 and 18°C. Parameters of the 2D multislice gradient echo (GRE) sequence were repetition time = 33.15 ms, echo time *TE* = 20 ms, flip angle = 14°, voxel size = 1.1×1.1×7.5 mm^3^, field of view = 170×170 mm^2^, bandwidth = 65 Hz/Pix and data acquisition time = 38 s. (Due to this protocol no radio frequency induced heating was noticed in bulb thermometer equipped phantoms at ambient temperature.).

Temperature induced phase shifts were obtained as differences between means of phase changes of RBC located around the thermometer tip during warm up and means of reference phantom phases [27]. A PRF thermal coefficient *α*_RBC_ of RBC was determined for each calibration measurement series from linear fit of resultant phase differences *φ* as function of temperature *T* between 1 and 18°C according to *α*_RBC_ = (*1/2π⋅γ⋅B_0_⋅TE*)⋅(*dφ/dT*), where *γ* is the gyromagnetic ratio of ^1^H nuclei, *B*_0_ the main magnetic field strength and *dφ/dT* the slope of the respective fit [26]. *α*_RBC_ mean value was used for calculation of temperature maps in RBC warm up experiments.

### PRF Thermometry of RBC Warm Up

To prevent uncontrolled thermal transfer processes and motion as well as to ensure a fast and reproducible experimental onset, RBC units were stored, transported and investigated in upright position in a two-layer plastic holder (Lego®) with minimum distance of 1 mm between frame and RBC. Withdrawn from storage, RBC units were plugged into an adjustment unit (Fig. 1b) equipped with 2 reference phantoms (kept at *T*_ambient_ throughout experiments) which was positioned in the MR system in the center of a 12 channel head coil. Immediately after exposition to ambient temperature (mean setup time = 16±3 s) reference and difference phase images were acquired with the multislice GRE sequence described for calibration measurements covering the entire RBC volume with equidistant sagittal slices (gap = 90–95%). After 20 measurements recorded with 3 min pause time (total investigation time = 69.7 min), turbo spin echo (TSE) images for segmentation were acquired in identical slice positions (repetition time = 3000 ms, echo time = 51 ms, flip angle = 170°, voxel size = 0.5×0.5×7.5 mm^3^, field of view = 170×170 mm^2^, bandwidth = 159 Hz/Pix, turbo factor = 5, number of slices = 7, data acquisition time = 3 min 17 s).

### Image Analysis

**Figure 2:**
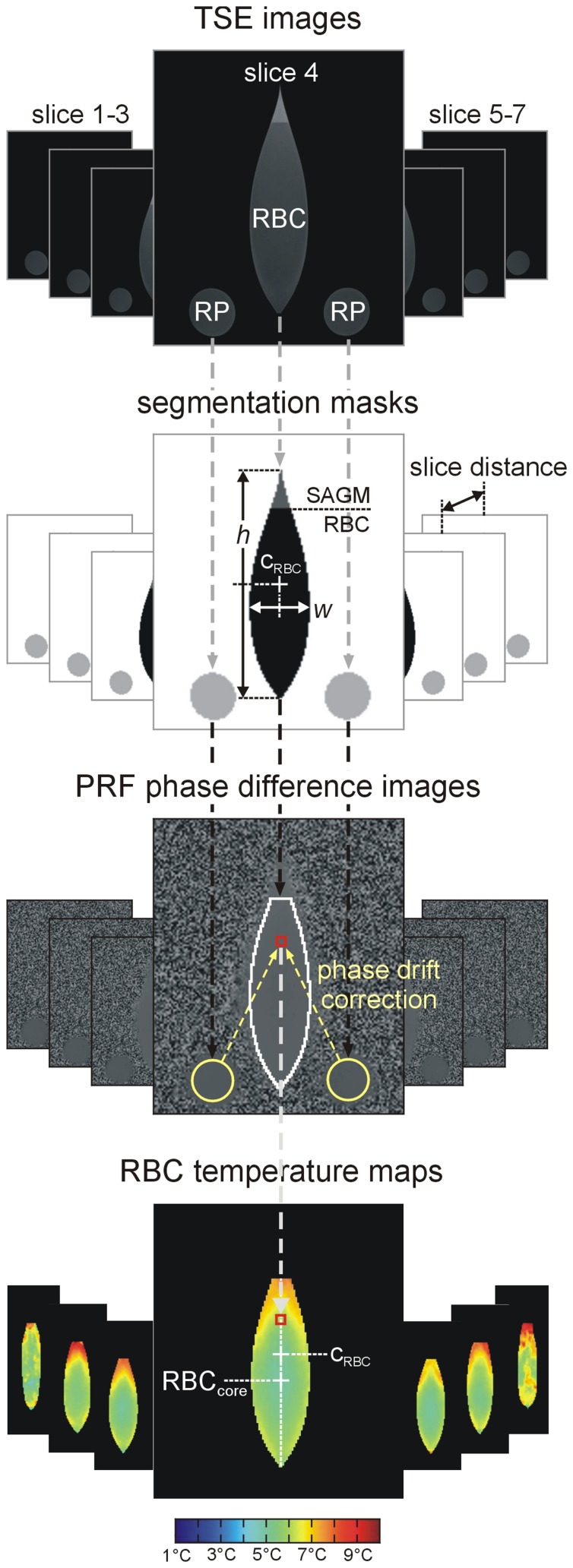
PRF image evaluation flow chart . TSE images were used to segment RBC volume, SAGM and reference phantoms RP. Sample height *h* and width *w* and center c_RBC_ = *h*/2 were determined from the central slice. RBC volume was calculated as sum of segmented RBC areas in the 7 slices multiplied by slice distance. After phase drift correction via medians of reference phantom phases, corrected RBC difference phases *φ* were recalculated to temperatures, which can be visualized color encoded. Thermal RBC core (RBC_core_) was defined as region with minimum temperature in the central slice throughout warm up. It should be noticed, that slices 1 and 7 are slightly noisier than the more central slices because of their close vicinity to the irregular borders of the pouch and the large echo time of 20 ms choosen to increase overall precision of temperature measurements.

Phase difference images were transformed to RBC temperature maps by dedicated software developed in Matlab (R2010b. The MathWorks Inc., Natick, Massachusetts). Evaluation flow chart, including RBC, SAGM and reference phantom segmentation, determination of non-temperature-related phase differences in the reference phantoms and correction of phase differences in RBC by the median of the phase differences in the reference phantoms is shown in Fig. 2. From corrected phase differences *φ* in RBC, corresponding temperatures *T* were calculated according to *T* = *T*_storage_+(*φ*/2*π⋅α*_RBC_ *⋅γ⋅B_0_⋅TE*) where *α*_RBC_ is the mean PRF thermal coefficient of RBC derived from calibration measurements and *T*_storage_ the temperature of RBC at onset of measurements [19]. RBC volume (as RBC area times slice distance), RBC pouch’s volume (as pouch’s area including RBC and SAGM times slice distance) height and width as well as its center position were derived from segmentation. Thermal RBC core position was defined as the position in the sample, where time-averaged temperature was minimal.

**Figure 3:**
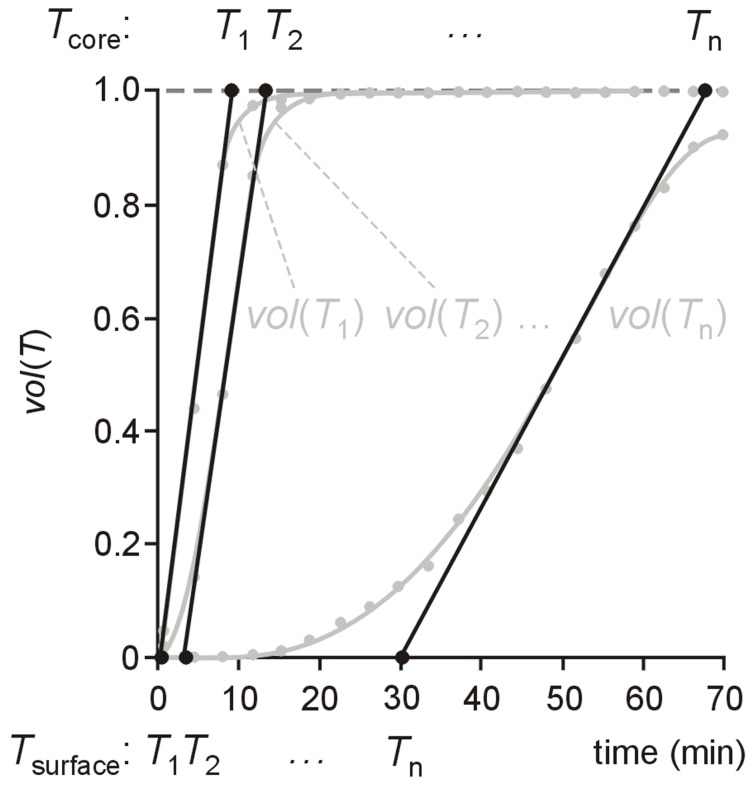
Determination of time courses of surface temperature and core temperature . For temperatures *T*_1_, *T*_2_, …, *T*_n_ time series of volume fractions *vol*(*T*_1_), *vol*(*T*_2_), …, *vol*(*T*_n_) were derived from time-resolved temperature maps and interpolated by cubic splines. Times when tangents at half maximum of the respective volume fraction crosses 0 are interpreted as the times, when surface temperature *T*_surface_ = *T*_1_, *T*_2_, …, *T*_n_, times when they cross 1 are interpreted as the times, when core temperature *T*_core_ = *T*_1_, *T*_2_, …, *T*_n_.

At each time temperature maps were employed to determine RBC mean temperature *T*_mean_ (calculated as average temperature of all voxels in the RBC volume) as well as RBC volume fractions *vol*(*T*) exceeding a temperature *T* for all integer-valued *T>T*_storage_. Linear extrapolation at half maximum of volume fraction-time curve *vol*(*T*) was used to specify (1) the time when RBC volume starts to exceed *T*, allowing *T* to be interpreted as surface temperature *T*_surface_ and (2) the time when 100% of RBC volume exceeds *T*, allowing *T* to be interpreted as core temperature *T*_core_. Evaluation of RBC volume fractions *vol*(*T*) for different temperatures *T* resulted in time courses of RBC surface and core temperatures (Fig. 3).

### Modeling Time Dependence of Mean, Surface and Core Temperature during Warm Up

For the analytical description of RBC’s warm up processes, it was hypothesized, that mean temperature *T*_mean_ during warm up is described by a lumped capacitance model of heat transfer [21], [22]. Consequently relative mean temperature difference *θ*_mean_ = (*T*_ambient_–*T*_mean_)/(*T*_ambient_–*T*_storage_) to ambient temperature T_ambient_ should fulfill
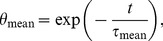
(1)where *t* is the time the RBC pouch is exposed to ambient temperature and *τ*_mean_ is a RBC pouch specific time constant, not dependent on storage temperature *T*_storage_ or difference between ambient and storage temperature *T*_ambient_–*T*_storage_, respectively. Moreover, for RBC surface and core temperatures it was hypothesized, that relative surface and core temperature differences *θ*_surface_ and *θ*_core_ to ambient temperature *T*_ambient_ follow “shifted” exponential decays. Thus, *θ*_surface_ and *θ*_core_ should fulfill
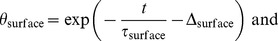
(2)

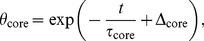
(3)with time constants *τ*_surface_ and *τ*_core_ and shifts Δ_surface_ and Δ_core_. The shifts characterize (in units of time constants) how much earlier (or later in case of core) the decay of *θ*_surface_ (and *θ*_core_) starts than the instantaneous decay of *θ*_mean_.

Relative temperature spread *θ*_spread_ in RBC was introduced according to *θ*_core_–*θ*_surface_, becoming maximal at
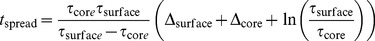
(4)


Moreover, by eliminating time, Eqs (1)-(3) can be transformed to
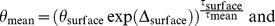
(5)

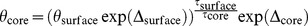
(6)relating relative surface temperature difference with relative mean and core temperatures.

### Statistical Analysis

Statistical analysis including linear fit of phase differences versus temperature for the calibration measurements as well as fitting of the exponential decays of the relative temperatures to the experimental RBC warm up data was performed using NCSS (Hintze J. (2007). NCSS. LLC. Kaysville, Utah). Mean values are given together with standard deviations. Standard deviations of fitted parameters were employed to calculate uncertainties of (relative) temperature (difference) courses via Gaussian law of error propagation. Dependency of time constants, shifts and (time of) maximal temperature spread on storage temperature and geometric parameters of the RBC pouches were investigated by means of correlation and linear regression analysis. *p*-values smaller 0.01 were considered as statistically significant.

## Results

### PRF Thermal Coefficient of RBC

Linear correlation between RBC phase differences and temperature changes in the calibration measurements was close to perfect (mean correlation coefficient 0.999±0.001). Mean PRF thermal coefficient of RBC was *α*_RBC_ = 1.05⋅10^−8^±0.02⋅10^−8^°C^−1^.

### Qualitative Description of RBC Warm Up Temperature Distributions

**Figure 4:**
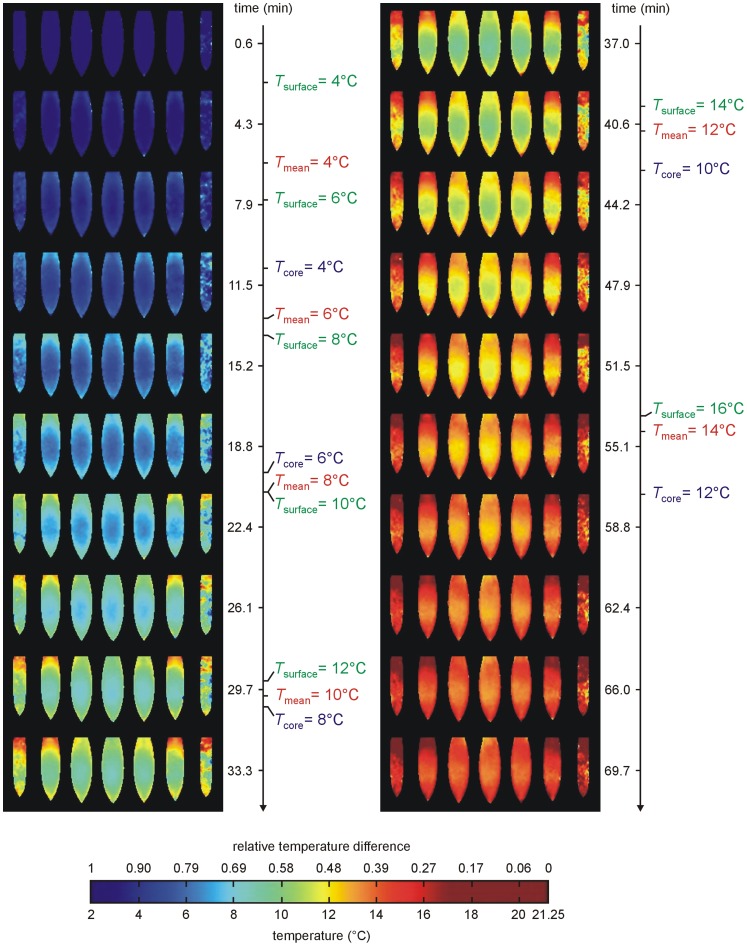
Typical RBC warm up temperature distribution. Color encoded time-resolved temperature maps of RBC withdrawn from 3.6°C storage temperature exposed to 21.25°C ambient temperature. Corresponding mean, surface and core temperatures are indicated.

Similar qualitative warm up phenomena were observed in all RBC units irrespectively of storage temperatures ranging from 1 to 6°C (mean *T*_storage_ = 3.6±1.4°C). A typical example of RBC temperature maps derived from PRF thermometry measurements is shown in Fig. 4.

Withdrawn from steady storage conditions, RBC’s temperature at the surface of the pouch and at SAGM border immediately started to rise. During warm up heat was continuously transferred from RBC surface to the adjacent inner layers, causing an increasingly non-uniform temperature distribution in the sample with isotherms located symmetrically around temperature core RBC_core_ in both transversal (horizontal and latitudinal) extensions of the unit. In vertical extension RBC_core_ was displaced by 13±4% from pouch center. Highest temperatures, the respective *T*_surface_, were reached at the surface at sections with smaller width at the top of the pouch.

Increases in mean, surface and core temperatures (or decreases of relative mean, surface and core temperature differences) decreased with increasing warm-up time.

### Time and “Geometry” Dependence of Mean, Surface and Core Temperatures during Warm Up

**Figure 5:**
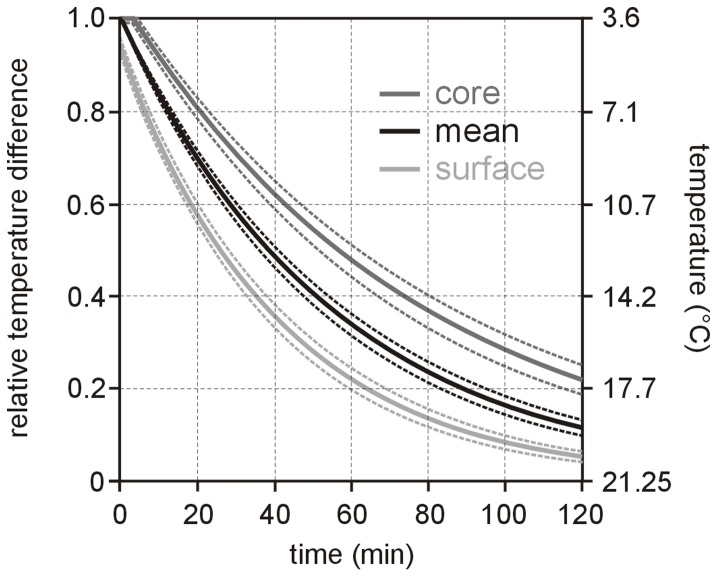
Time dependence of relative temperatur differences. Average time courses (solid lines) and corresponding uncertainties (dotted lines) of relative temperature differences of mean, surface and core temperature to ambient temperature of RBC during warm up. Recalculation of relative temperature difference scale to temperature scale at the right hand side was done (for convinience) for *T*_storage_ = 3.6°C and *T*_ambient_ = 21.25°C.

Measured relative temperature differences *θ*_mean_, *θ*_surface_ and *θ*_core_ fulfilled lumped capacitance model of heat transfer [Eq. (1)] and “shifted” exponential decays [Eqs (2) and (3)] close to perfect. Mean *R* ^2^ were 0.999±0.001, 0.996±0.004 and 0.998±0.002, respectively. The resulting mean time constants were *τ*_mean_ = 55.3±3.7 min, *τ*_surface_ = 41.4±2.9 min and *τ*_core_ = 76.8±7.1 min, resulting mean time shifts Δ_surface_ = 0.07±0.02 and Δ_core_ = 0.04±0.01. Average time courses of *θ*_mean_, *θ*_surface_ and *θ*_core_ together with corresponding uncertainties are shown in Fig. 5. Mean time when relative temperature spread is maximal [Eq. (4)] was *t*_spread_ = 45.5±4.1 min, the corresponding mean maximal relative temperature spread was *θ*_spread_(*t*_spread_) = 0.26±0.04.

**Figure 6:**
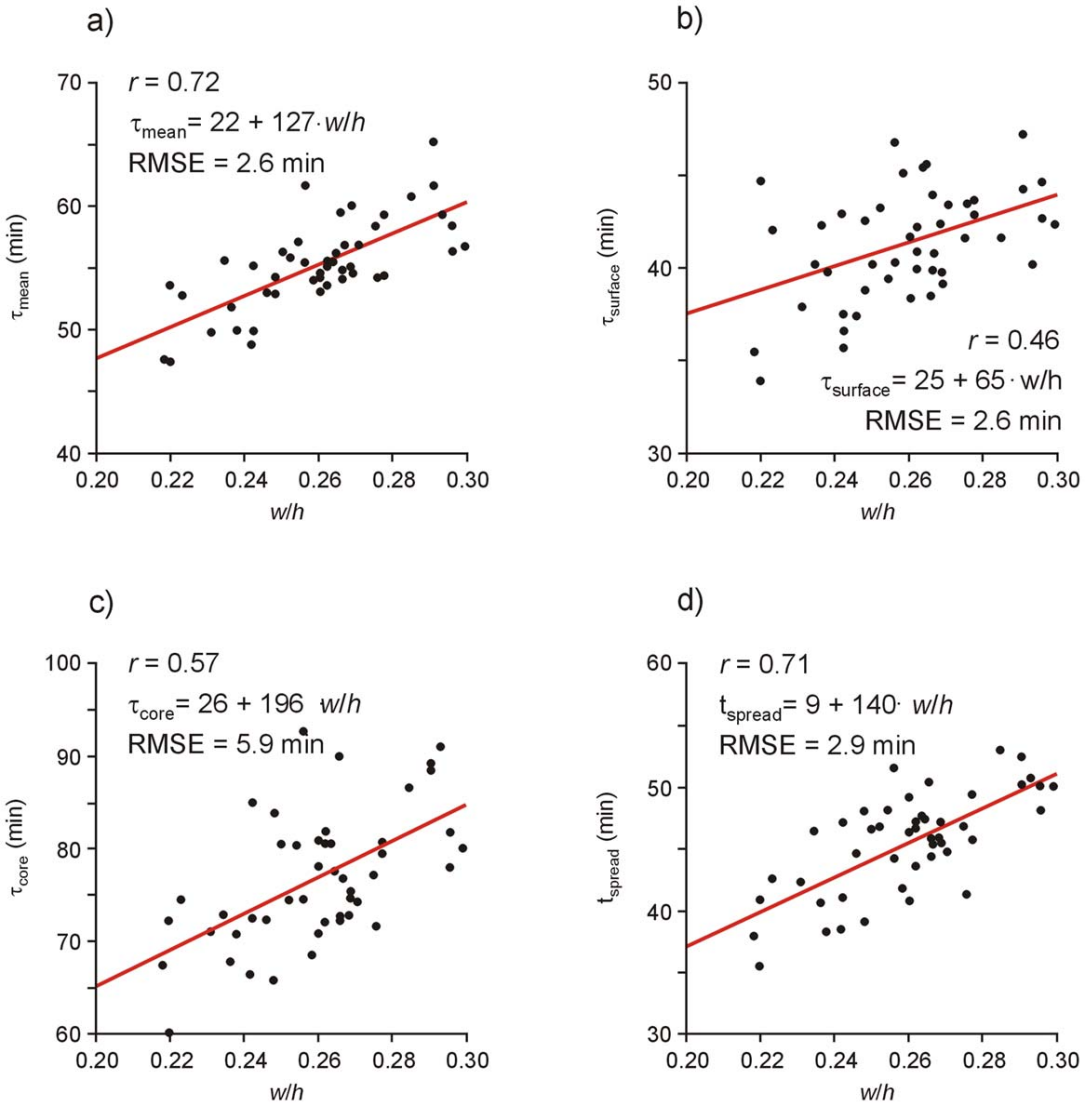
Time constants and time of maximum temperature spread versus width-length-ratio. Scatterplots and linear regression lines of time constants (a) *τ*_mean_, (b) *τ*_surface_, (c) *τ*_core_ and of (d) time *t*_spread_ of maximal temperature spread versus the width-height-ratio *w/h* of RBC pouches. Regression equations are to be understood in minutes, *RMSE* denotes root mean square error, *r* Pearson’s correlation coefficient.

Neither time constants and shifts nor (time of) maximal relative temperature spread depended on storage temperature or *T*_ambient_–*T*_storage_. Variations in geometric parameters RBC volume (255±17 ml), pouch volume (287±26 ml), pouch height *h* (13.1±0.5 cm) and width *w* (3.4±0.2 cm) were small and shifts as well as maximal relative temperature spread did not depend on these parameters (except a weak but significant correlation of *r* = −0.47 between Δ_surface_ and pouch volume). Correlations between time constants and time of maximal temperature spread with geometric parameters are summarized in Table 1. Linear regression results of *τ*_mean_, *τ*_surface_, *τ*_core_ and *t*_spread_ versus the width-height-ratio *w/h* are shown in Fig. 6.

**Table 1:** Correlation coefficients between time constants and time of maximal temperature spread with geometric RBC pouch parameters.

Geometric parameter	*τ*_mean_	*τ*_surface_	*τ*_core_	*t*_spread_
RBC volume	0.39* a	0.17	0.40*	0.41*
Pouch volume	0.59*	−0.02	0.46*	0.55*
RBC-to-pouch-volume-ratio	−0.38*	0.18	−0.23	−0.34
Height *h*	−0.50*	−0.39*	−0.32	−0.47*
Width *w*	0.68*	0.38*	0.59*	0.68*
Width-height-ratio *w/h*	0.72*	0.46*	0.57*	0.71*

a *indicates statistical significance.

### Temperature Prediction from Surface Temperature

Mean quotients of time constants *τ*_surface_/*τ*_mean_ and *τ*_surface_/*τ*_core_ appearing in Eqs (5) and (6) were 0.75±0.06 and 0.54±0.06, respectively. Both did not depend significantly on storage temperature and *T*_ambient_–*T*_storage_. Significant correlations with geometric parameters of RBC pouches were found for pouch volume (*r* = −0.53 in case of *τ*_surface_/*τ*_mean_, *r* = −0.40 in case of *τ*_surface_/*τ*_core_).

**Figure 7:**
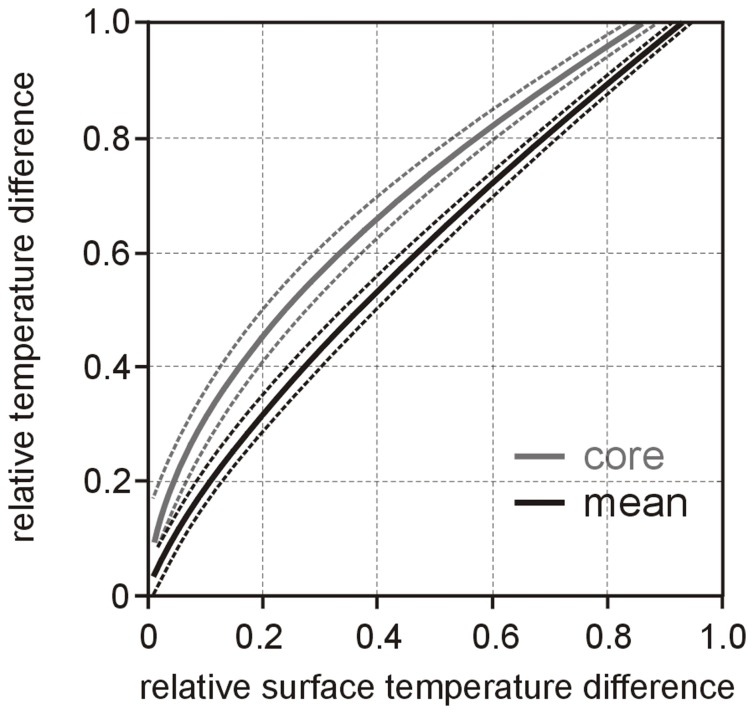
Prediction of relative mean and core temperatures from relative surface temperature. Dependence of relative mean and core temperature differences *θ*_mean_ and *θ*_core_ on relative surface temperature differences *θ*_surface_ (solid lines) together with corresponding uncertainties (dotted lines) during warm up.

**Figure 8:**
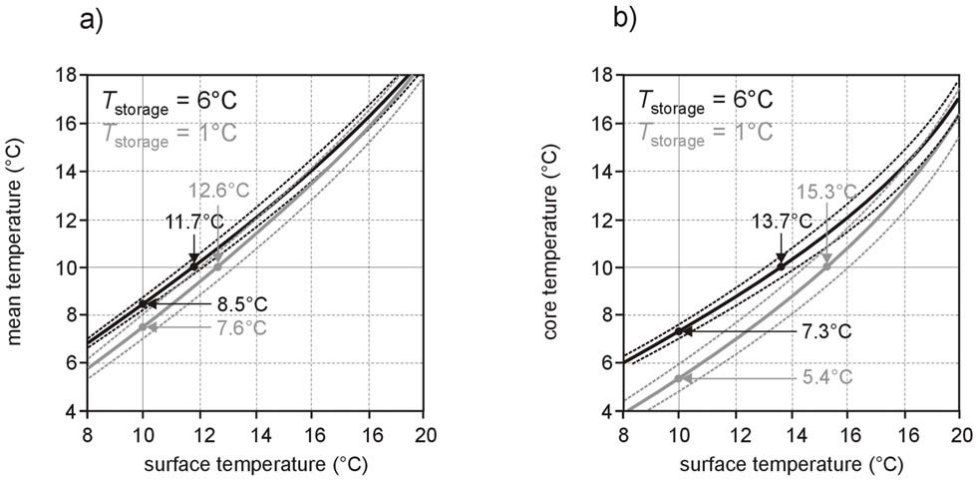
Influence of storage temperature on the prediction of mean and core temperatures from surface temperautre. Dependence of (a) mean temperature *T*_mean_ and (b) core temperature *T*_core_ on surface temperature *T*_surface_ (solid lines) together with corresponding uncertainties (dotted lines) for the “extreme” storage temperatures *T*_storage_ of 1°C and 6°C and ambient temperature *T*_ambient_ = 21.25°C. Mean values and standard deviations of respective quotients of time constants and shifts were used for calculations as these quantities did not depend significantly on storage temperature. Results for surface, mean and core temperatures of 10°C are emphasized (solid lines).

Average dependencies of relative mean and core temperature differences *θ*_mean_ and *θ*_core_ on relative surface temperature difference *θ*_surface_ together with corresponding uncertainties are shown in Fig. 7. The influence of storage temperature *T*_storage_ on the prediction of absolute mean or core temperature from absolute surface temperature is indicated in Fig. 8.

## Discussion

Time-resolved temperature distributions of RBC units removed from refrigerated storage between 1 and 6°C and exposed to ambient temperature of 21.25°C assessed non-invasively by PRF thermometry were visualized and analyzed by dedicated software. Major findings derived from this study were (1) RBC mean temperature time course during warm up almost perfectly followed a lumped capacitance model law, (2) surface and core temperature warm up time courses of RBC almost perfectly fulfilled time-shifted exponential models and (3) neither time constants nor shifts depended significantly on storage temperature or the difference between ambient and storage temperatures, which supports the general applicability of the analytical description and corresponding RBC constants for arbitrary storage and ambient temperatures.

MR thermometry is typically employed for in vivo monitoring of temperature during thermotherapy-heating of tissue [18], [20], [27], [28], [29], [30]. Calibration experiments confirmed that PRF method, implying linear relationship between temperature and phase changes, is applicable to measure RBC temperature in the range between 1 and 18°C. PRF thermal coefficient *α*_RBC_ derived within the present study of RBC was close to the one of water [31] and in the range of other biological tissue [19], [27].

Removed from refrigerated storage to ambient temperature, RBC surface immediately starts to warm up while temperature in the core of the sample rises time delayed to peripheral layers causing non-homogeneous temperature distributions in RBC. As the average temperature spread in RBC reaches more than a quarter of the difference between ambient and storage temperature, which is consistent with results derived from invasive measurements [14], RBC’s resistance to heat transfer is not negligible and specification of the temperature assessed is inevitable.

Aside from mean temperature, which represents the temperature after “gentle mixing”, surface and core temperatures were introduced essentially noise and partial volume independent (Fig. 3) as maximal and minimal temperature in RBC. As highest temperatures appeared at the RBC surface (Fig. 4) the term surface temperature for maximal temperature is justified. Temperatures at RBC pouch’s surface were, however, inhomogeneously distributed as previously demonstrated by Johnson et al [10]. Surface temperature therefore represents the upper temperature bound reached at thinner layers of the pouch. RBC core temperature, required to maintain below 10°C during transport by current guidelines [5], [6], [8], was reached vertically displaced from the pouch center towards thicker sample layers. This might explain discrepancies in core temperature time courses described in previous studies applying thermocouple data loggers [1], [9], [14], [32].

Time dependence of relative mean, surface and core temperature differences to ambient temperatures of all samples were very well described by (shifted) exponential decays. Time constants and shifts did not depend significantly on the difference between ambient and storage temperature. Consequently lumped-capacitance-model-like formulas Eqs. (1)–(3), together with the mean values of the fitted constants (Fig. 5), should provide suitable descriptions of RBC warm up for wide ranges of not only storage but also ambient temperatures. Standard deviations of time constants and shifts as well as derived uncertainties of the relative temperature difference-time-curves shown in Fig. 5 are not solely caused by measurement errors, but result also from deviations in RBC unit’s geometry. Although variations in volume, height, width and width-height-ratio were small, all time constants correlated significantly with these geometric parameters. In accordance with Perry et al. [9] core (and mean) temperature time constants increased with RBC and pouch volume, confirming that more voluminous samples need longer to warm up. However, the correlation of all time constants with RBC width and width-height-ratio was stronger. This behavior is to be expected from lumped capacitive model of heat transfer, because time constants are proportional to body volume per heat transfer or pouch surface area [21], which should in turn be estimated from RBC pouch width, or even better, from unit’s width-height-ratio.

According to lumped-capacitance-model-like formulas Eqs. (1)–(3), times when absolute mean, surface or core temperatures of RBC during warm up exceed a temperature limit strongly depend on storage and ambient temperature. For example keeping RBC outside from refrigerated storage for less than 30 min thus ensures RBC core temperature to stay below 10°C only within certain ranges of storage and ambient temperatures: The mean relative core temperature difference to ambient temperature after 30 min was 0.70, which transforms to limit ambient temperatures of 31 and 20°C for storage temperatures of 1 and 6°C such that core temperature stays below 10°C. These limit temperatures support validity and applicability of the “30 minutes rule”. As for storage and ambient temperature of 1 and 20°C respectively, 10°C RBC core temperature is reached in mean only after 52 min, current guidelines could eventually allow longer “reuse-phase” of RBC units.

For the use of temperature sensitive labels attached to the surface of RBC pouch the problem transforms to the question if “inside” RBC core temperature can reliably be predicted from RBC surface temperature. Recently it could be shown that times when surface, mean and core temperatures reach 10°C during warm up tremendously differ but can be correlated with each other [15]. Eqs (5) and (6) as well as Fig. 7 provide the general relationship between mean, core and surface temperatures during RBC warm up. The sole appearance of quotients of time constants renders relationships to large extent independent from RBC geometry. However, prediction of mean and core temperature from surface temperature depends on storage and ambient temperature. Therefore precise temperature monitoring by temperature sensitive labels attached to the surface of RBC pouch is possible only if storage and ambient temperatures are known as well. The impact of the “RBC storage temperature memory effect” is shown in Fig. 8 by specifying mean and core temperature relations on surface temperature for recommended lowest (1°C) and highest (6°C) storage temperature limits for an ambient temperature of 21.25°C.

Regarding the present study, several limitations should be mentioned. RBC warm up during fast setup (16 s ±3 s) was not taken into account and mixing of RBC prior to MR measurement was not possible as residual motion would affect PRF thermometry. Furthermore storage temperature was not measured directly from entire samples but derived from RFID data logger measurements mounted in a reference RBC unit as internal probe continuously kept at storage temperature throughout experiments.

Limited measurement time (70 min) and time resolution (3 min) of PRF measurements were chosen to minimize B_0_ drifts and warming due to radio frequency absorption and to keep the total scan time reasonable. As temperature changes were rather slow an increase in time resolution should not add additional information, behavior of warm up temperatures above 70 min warm up time is, an extrapolation, which is not proven by the experiments.

Finally it has to be addressed, that there is an enormous manufacturer variability in geometry, material and properties of red blood cell containers and the present results apply a priori only to MacoPharma blood collection systems used in the study.

In conclusion, lumped capacitive model of heat transfer and shifted exponential decays represent simple analytical formulas to describe transient mean, surface and core temperatures of RBC during warm up, which might be a helpful tool in RBC temperature monitoring and quality control. Independence of constants on differences between ambient and storage temperature suggests validity of models for arbitrary storage and ambient temperatures.
